# *In Vivo* Antimalarial Activity of Crude Fruit Extract of *Capsicum frutescens* Var. Minima (Solanaceae) against *Plasmodium berghei*-Infected Mice

**DOI:** 10.1155/2020/1320952

**Published:** 2020-08-25

**Authors:** Getu Habte, Solomon Assefa

**Affiliations:** ^1^Department of Pharmacology and Clinical Pharmacy, School of Pharmacy, College of Health Sciences, Addis Ababa University, P.O. Box 1176, Addis Ababa, Ethiopia; ^2^Department of Pharmacy, College of Health Sciences, Mettu University, P.O. Box 318, Mettu, Ethiopia

## Abstract

**Background:**

The alarming spread of parasite resistance to current antimalarial agents is threatening malaria controlling efforts. This, consequently, urged the scientific community to discover novel antimalarial drugs. Successful and most potent antimalarial drugs were obtained from medicinal plants. *Capsicum frutescens* is claimed to possess an antiplasmodial activity in Ethiopian and Ugandan folkloric medicine. However, there is a lack of pharmacological evidence for its antiplasmodial activity. This study, hence, was aimed at evaluating the *in vivo* antiplasmodial activity of *C. frutescens* in a mouse model.

**Methods:**

The dried fruits of the plant were extracted with 80% methanol using cold maceration. A 4-day suppressive test was employed to ascertain the claimed antiplasmodial effect of the plant. Following inoculation with *P. berghei*, mice in treatment groups were provided with three dose levels (100, 200, and 400 mg/kg) of the extract, while 2% Tween 80 and chloroquine served as the negative and positive controls, respectively. Weight, temperature, packed cell volume, parasitemia, and survival time were then monitored.

**Results:**

The acute oral toxicity study revealed that the crude extract caused no mortality and revealed no overt sign of toxicity. In the 4-day suppressive test, all dose levels of the extract were found to exhibit a significant (*p* < 0.05) inhibition of parasitemia compared to those of the negative control. Maximum parasite suppression (93.28%) was exerted by the highest dose (400 mg/kg/day) of extract. Also, the extract significantly (*p* < 0.05) prolonged survival time and prevented body weight loss and reduction in temperature and anemia compared to the vehicle-treated group.

**Conclusion:**

This investigation found strong evidence that the fruit extract of *C. frutescens* is endowed with promising antiplasmodial activity. Hence, the plant could serve as a potential source of a newer antimalarial agent.

## 1. Background

Malaria is still causing significant mortality and morbidity in the global community [1]. More than half of the world population is estimated to be infected by the disease [2]. The sub-Saharan Africa is the most affected area. Of the admissions and outpatient visits to the health facilities, malaria is the number one disease of concern in this region. Furthermore, malaria deaths in this area account for more than 90% of the global deaths due to the disease [1, 2]. In the same way, the Ethiopian people are also at greater risk of contracting the disease. More than 75% of the country's people reside in areas that are highly prone to malaria [3–5].

Compared to adults, pregnant women and children below five years of age are more affected [6–9]. The disease not only causes mortality and morbidity but also poses socioeconomic crisis [1, 2, 4]. Expenses for hospitals, absence from schools, reduced work force, influences on tourism, and reduced attraction for investors are among the economic burdens from the disease. During the last decade alone, international partners and the malaria endemic country governments spent more than nineteen billion United States dollars in order to combat malaria [2–4].

Among the challenges to confront the disease, development of resistant strains of the parasite to the conventional antimalarial drugs is a common phenomenon. In other words, the plasmodial parasites that were initially responding to the antimalarial drugs are now losing sensitivity [10]. Even now, there are warning findings regarding the resistant strains of the parasite to the most effective drugs of today, artemisinins, in Southeast Asia and Africa [11, 12]. In fact, there is high probability that these resistant types can move to the rest of the world and be a global threat. This issue necessitates the scientific community to research for new antimalarial agents having better safety and efficacy [11–13].

As history shows, plants were a major source for the antimalarial drugs. This further hints at investigating medicinal plants to discover novel antimalarial drugs [14–16]. Medicinal plants are widely utilized to treat malaria where the disease is endemic. For the treatment of malaria, more than 160 plant families are documented as folk medicines [17]. Several African plants are scientifically proven to possess antimalarial properties [18]. *Capsicum frutescens* var. minima has a claimed folkloric medicinal value in the management of malaria and other diseases in Africa [19, 20]. Nonetheless, there is lack of scientific evidences that verified the claimed antiplasmodial activity of this plant in experimental animals. Thus, this study investigated the *in vivo* antiplasmodial activity of *C. frutescens*, the variant commonly used in Sasiga District of Ethiopia as an antimalarial remedy, against chloroquine-sensitive *Plasmodium berghei* (ANKA) infection in mice.

## 2. Materials and Methods

### 2.1. Collection of Experimental Plant

For the purpose of authentication, first, the branch of *C. frutescens* with its leaves and fruits attached were collected in January 2018, from Sasiga District, west Ethiopia. Further collection of the plant fruits was done for use in the extraction process. A plastic bag was used to carry the fruits during transportation. The plant was identified and authenticated by a taxonomist at the National Herbarium, Addis Ababa University, College of Natural and Computational Sciences. After giving a code of GH 02/2018 to the plant voucher specimen, for future reference, the material was deposited in the herbarium.

### 2.2. Preparation of the Crude Extract

In order to remove dust from the fruits, the fruits were first properly washed with distilled water and cleaned with a muslin cloth. To make a coarse powder and facilitate an extraction process, the plant fruits were air dried under shade at room temperature and pulverized with the aid of a mortar and pestle. The cold maceration technique as per the method described by Bello et al. [19] was then utilized to prepare the 80% methanolic crude extract of the fruit. Accordingly, 500 g of coarse powder was soaked in 750 ml of 80% methanol solution using an Erlenmeyer flask. A mechanical shaker (Bibby Scientific Limited, Stone, Staffordshire, UK) operating at 120 rpm for 72 hours was used to facilitate the extraction process.

At the end of the maceration, the flask was removed out of the shaker, put on a table, and left for 30 minutes to form a layer of distillate and marc. Then, separation of the marc from the resulting distillate containing the 80% methanol crude extract was done first using muslin cloth, followed by filtration with the aid of a Whatman filter paper number 1 (Whatman®, England) under suction filtration. This cold maceration procedure, in the same solvent system, was repeated twice using the marc left. The filtrate was then transferred to a round bottom flask and concentrated using a rotary evaporator (Buchi Rotavapor R-200, Switzerland) under reduced pressure. Then, the resulting extract was frozen in a deep freezer overnight and freeze dried with a lyophilizer (Operon, Korea Vacuum Limited, Korea) to remove water at -50°C and vacuum pressure of 200 mbar. The concentrated extract was transferred into an amber glass bottle and kept at -20°C until use. The percentage yield was calculated using the following formula:

(1) $$\begin{array}{c} \text{Pecentage yield}=\frac{\text{weight of dried extract}}{\text{weight of pulverized fruit used in maceration}}*100. \end{array}$$

### 2.3. Experimental Animals

The experiment was conducted on Swiss albino mice whose weights were in a range between 25 and 31 g and 6 to 8 weeks old. The animals were obtained from the Ethiopian Public Health Institute. Before conducting the experiment, acclimatization of the animals for a week to the experimental condition was done. The experiment was conducted in an animal house at Addis Ababa University, College of Health Sciences, School of Pharmacy. Polypropylene cages were utilized to house the animals where they stayed in a 12-hour light-dark cycle. A standard pellet food was given regularly to the mice while water was provided *ad libitum*. The standard animal handling practice utilized in the present study was following a well-accepted animal handling protocol [21].

### 2.4. Acute Toxicity Test

To determine the safety of the plant extract, the Organization for Economic Cooperation and Development number 425 guideline [22] was used to evaluate the oral acute toxicity profile. In this test, after weighing a single overnight-fasted mouse, 2000 mg/kg of the crude extract was provided to the mouse using oral gavage at once. Then after, food was withheld for 2 hours but not water. The mouse was then observed continuously for the first 30 min and intermittently for 4 h, over a period of 24 h. As neither death nor any signs of acute toxicity were seen, similar dosage of the extract was provided to another four female mice. Then, the mice were followed for 2 weeks to see if changes that are signs of acute toxicity occur.

### 2.5. *In Vivo* Antimalarial Screening

#### 2.5.1. Grouping and Dosing of Animals

As mentioned in Section 2.3, Swiss albino mice were utilized for evaluating the *in vivo* antiplasmodial effect of the 80% methanolic crude fruit extract of *C. frutescens*. Thirty male mice were intraperitoneally infected with *P. berghei* and randomly divided into five groups of six mice each, in separate cages. The negative control received 10 ml/kg dose of solvent for reconstitution (Tween 80 2% *v*/*v*). Three of the groups received different dose levels of the extract (100 mg/kg, 200 mg/kg, and 400 mg/kg). Mice in the last group (positive control) were treated with 10 mg/kg dose of chloroquine (CQ) base. Dose selection for the extract was determined based on the result of acute toxicity, and preliminary screening test done on the plant extract.

#### 2.5.2. Parasite Inoculation

Chloroquine sensitive *P. berghei* (ANKA strain) was obtained from the Ethiopian Public Health Institute. The parasites were then maintained by serial passage of blood from infected mice to noninfected ones every week until 30-37% of the parasitemia level was attained [23]. The parasitemia levels of the donor mice were determined from the blood collected by cutting a 0.5 to 1 mm section from the tail of the mice with scissors. Mice with the aforementioned level of rising parasitemia were used as donors. Donor mice were euthanized using halothane in a closed chamber, and the infected blood was immediately collected through cardiac puncture into a heparinized vacutainer tube [24- 26]. The collected blood from all donor mice was pooled together to avoid variability and then diluted in normal saline [24]. The blood was then diluted with physiological saline (0.9%) in such a way that the final suspension would contain about 1 × 10^7^ parasitized red blood cells (PRBCs) in every 0.2 ml of blood [25]. Experimental animals were inoculated with 0.2 ml of diluted blood, intraperitoneally.

#### 2.5.3. The 4-Day Suppressive Test

The method described by Peter et al. [26] was utilized to carry out the *in vivo* schizontocidal activity of the extract against early chloroquine-sensitive *P. berghei* infection. On the first day (D_0_), an apparently healthy mouse in each group was coded and inoculated with 0.2 ml of blood containing about 1 × 10^7^*P*. *berghei*-infected RBCs. Then, three hours after infection (D_0_), each animal in each group was weighed and orally provided with the corresponding dosage of the extract and the controls using oral gavage for four consecutive days (D_0_-D_3_). At the beginning of D_0_ and/or at the end of the 4^th^ day (D_4_), the parameters detailed below were determined and the mice were monitored daily for 30 days to evaluate their survival time [26].

#### 2.5.4. Determination of Parasitemia and Survival Time

At D_4_, the blood was collected from the tail of each mouse using clean and nongreasy slides to prepare thin blood films. The slides were stained with freshly prepared 10% Giemsa for 15 minutes. The stain was then washed off with distilled water. After allowing the slides to air-dry, the slides were viewed microscopically using the ×100 objective. By counting the number of PRBCs out of erythrocytes in random fields of the microscope, the percentage parasitemia (PP) was obtained. For each mouse, two stained slides were examined. Three fields with approximately 200-500 cells were counted for each slide, and PP for each mouse was determined as described below [27, 28]:

(2) $$\begin{array}{c} \text{PP}=\frac{\text{PRBC}}{\text{total number of RBCs counted}}\times 100. \end{array}$$

The following formula was used to calculate the mean percentage parasitemia suppression (PPS) [25, 28]:

(3) $$\begin{array}{c} \text{PPS}=\frac{\text{mean PP in negative control}-\text{mean PP in treatment group}}{\text{mean PP in negative control}}\times 100. \end{array}$$

For each group, the mean survival time (MST) was calculated as follows [16]:

(4) $$\begin{array}{c} \text{MST}=\frac{\text{sum of survival time of all mice in a group }\left(\text{days}\right)}{\text{total number of mice in that group}}. \end{array}$$

#### 2.5.5. Determination of Weight, Temperature, and Packed Cell Volume

The weight and rectal temperature of each mouse were recorded just before treatment and after treatment on D_4_. The mean percentage changes were then calculated and analyzed for each group [16, 24]. In the same way, packed cell volume (PCV) was measured before inoculation and after treatment. The blood was collected from the tail of each mouse in heparinized microhematocrit capillary tubes to determine PCV. The capillary tubes were filled to 3/4^th^ of their height with blood and sealed with sealing clay at their dry end. The tubes were then placed on a microhematocrit centrifuge (Centurion Scientific, UK) with the sealed end facing the periphery and centrifuged at 11,000 rpm for 5 minutes [29]. Eventually, PCV was determined using the standard hematocrit reader (Hawksley and Sons, England) according to the formula indicated below [27, 28]:

(5) $$\begin{array}{c} \text{PCV}=\frac{\text{volume of erythrocyte in a given volume of blood}\;}{\text{total blood volume examined}}\times 100. \end{array}$$

### 2.6. Phytochemical Screening

The phytochemical constituents of the 80% methanolic crude fruit extract of *C. frutescens* were investigated qualitatively and quantitatively following standard methods [30–32]. The major plant phytochemicals including alkaloids, saponins, tannins, flavonoids, glycosides, steroids, and terpenoids were assayed.

### 2.7. Data Analysis

After organizing, the data were fed into SPSS version 22 and then analyzed. To compare the mean PPS, MST, changes in mean body weight, PCV, and rectal temperature of the *P*. *berghei*-infected mice between the extract received groups and the controls and among the extract received groups, one-way analysis of variance (ANOVA) followed by *Tukey post hoc* test was done. *p* value less than 0.05 was considered to be statistically significant with analysis at 95% confidence interval.

## 3. Results

### 3.1. Percentage Yield of the Extract

The physical nature of the extract was found to be dry brownish powder. A total of 97.9 g (19.58% yield) of the extract was harvested from the 80% methanolic crude extract of the fruits of *C. frutescens*.

### 3.2. Acute Toxicity Test

The test for acute toxicity showed that no mortality was observed within the first day and the next 2-week period of observation. Furthermore, the plant caused no visible signs of acute toxicity as evidenced by the gross behavioral and physical observations of the experimental mice.

### 3.3. The 4-Day Suppressive Test

#### 3.3.1. Effect on Parasitemia and Survival Time

The chemosuppressive effect of the plant extract is summarized in Table 1. All dose levels of the crude fruit extract evaluated in the study exhibited a statistically significant (*p* < 0.05) difference in reducing the parasite load compared to the negative control, in a dose-dependent fashion. The 400 mg/kg/day dose of the extract exhibited the highest parasitemia inhibition (93.28%) compared to other doses. Nevertheless, the effect produced by the crude extract was inferior to the standard drug, which cleared the parasite to an undetectable level. In addition, the longest mean survival time (27.42 days) was exhibited at the highest dose administered. CQ-treated groups, on the other hand, survived throughout the monitoring period (>30 days).

#### 3.3.2. Effect on Body Weight and Rectal Temperature

As compared to the vehicle-treated group, the three doses of the extract significantly (*p* < 0.05) averted body weight loss. Compared to the negative control group, moreover, all the dose levels of the extract were able to significantly prevent body temperature dropping due to parasite infection. This protection against body weight loss and rectal temperature dropping was dose-dependent. Accordingly, the highest dose given, 400 mg/kg/day (Table 2), showed the highest protection against both weight and temperature reduction.

#### 3.3.3. Effect on Packed Cell Volume

**Figure 1:**
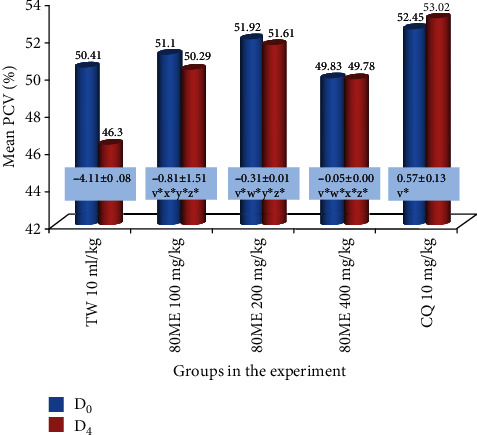
Changes in packed cell volume of mice infected with *P. berghei* and treated with the 80% methanolic crude fruit extract of *Capsicum frutescens*. Values are described as mean ± SEM (*n* = 6). D_0_: pretreatment value on day 0; D_4_: posttreatment value on day four; TW: 2% Tween 80; CQ: chloroquine base; 80ME: 80% methanol extract; PCV: packed cell volume. ^v^Compared to TW 10 ml/kg; ^w^compared to 100 mg/kg; ^x^compared to 200 mg/kg; ^y^compared to 400 mg/kg; ^z^compared to CQ10; ^∗^*p* < 0.05. The numbers in rectangles shown across the graph represent the difference in mean PCV between D_0_ and D_4_.

As compared to the vehicle-treated mice, the mice in the extract-treated group exhibited a dose-dependent and statistically significant (*p* < 0.05) protection against reduction of PCV. Furthermore, the upper dose (400 mg/kg/day) of the extract showed the highest protection against reduction of PCV (Figure 1). The rank order of protection from infection-induced reduction in PCV was 10 mg/kg dose of CQ > 400 mg/kg of extract > 200 mg/kg of extract > 100 mg/kg of extract.

### 3.4. Phytochemical Screening

#### 3.4.1. Preliminary Qualitative Phytochemical Test

Preliminary phytochemical screening test done on the 80% methanolic crude fruit extract of *C. frutescens* revealed that the plant contains alkaloids, saponins, tannins, flavonoids, glycosides, and terpenoids. However, steroids were absent (Table 3).

#### 3.4.2. Quantitative Phytochemical Investigation

**Figure 2:**
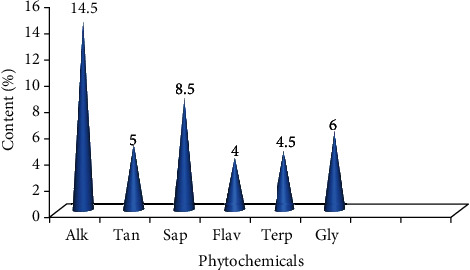
Quantitative determination of phytochemicals in the 80% methanolic crude fruits extract of *Capsicum frutescens*. Alk: alkaloids; Tan: tannins; Sap: saponins; Flav: flavonoids; Terp: terpenoids; Gly: glycosides.

The study to determine the amount of each phytochemical in the 80% methanolic fruit crude extract of *C. frutescens* depicted that alkaloids were the most abundant followed by saponins (Figure 2).

## 4. Discussion

More than 80 percent of the African population rely on traditional herbal medicine to meet their primary healthcare demand. Nonetheless, pharmacological investigations to standardize and set the antimalarial efficacy and safety of such plants were not largely done to this indigenous gift [3, 5]. *C. frutescens* is commonly used as a folk medicine for malaria treatment. Consequently, this study investigated the *in vivo* antimalarial activity of the plant in an attempt to contribute to the discovery of novel antimalarial drugs using a 4-day suppressive test [19, 20]. The test is a widely utilized standard *in vivo* antimalarial screening model in scientific research. This is mainly because the *in vivo* study takes into account the possible prodrug effect and involvement of the immune system in killing the parasites towards the efficacy and safety of the plant under investigation [27].

In the present study, crude fruit extract of *C. frutescens* showed a dose-dependent significant inhibition of parasitemia compared to the vehicle-treated group. This is comparable with similar studies done on *Croton macrostachyus* [29] and *Withania somnifera* [33]. However, chemoprevention efficacy of the plant is superior to our previous report on *Schinus molle* [16].

Phytoconstituents such as alkaloids, abundantly localized in the plant extract, could be responsible for the antiplasmodial activity as plant-derived alkaloids such as quinine are evidenced to possess a potent antimalarial activity. This plant also contains terpenoids, phenolic compounds, and flavonoids. These bioactive principles have been reported to possess a range of therapeutic activities including antimalarial activity in the literature [34, 35]. The chemosuppressive effect exerted could be via indirect boosting of the immune system or inhibition of other target pathways which are not fully realized. The phytosteroids, phenolic compounds, and flavonoids observed in this plant have been proven to possess potential immunomodulatory, anti-inflammatory, and antioxidant activities. Furthermore, the plant constituents may target the previously discovered targets in the pathogenesis and life cycle of the malaria parasite but with a unique or similar mechanism of action [34–38].

In discovering antimalarials from plants, the extract is ideally expected to prevent reduction in PCV, body weight, and body temperature due to the development of parasitemia [25]. Though a significant prevention of body weight loss was exhibited by crude extract, the mean value of the body weight showed reduction in treatment groups on D_4_ as compared to D_0_. This could be ascribed to the inability of the extract to completely eradicate the parasite load [16, 30]. PCV was monitored to assess the ability of the plant in ablating malaria-induced hemolysis. The lowest dose exhibited the least protection against hemolysis compared to the other two upper doses. This could be perhaps because of the abundant phytoconstituents concentrated in the upper doses.

The three doses of methanolic crude extract significantly prevented rectal temperature drop due to parasitemia as compared to the vehicle. These activities probably indicate that the extract ameliorates some pathological processes that cause reduction in internal body temperature and metabolic rates. In a 4-day suppressive test, a candidate antimalarial agent should prolong survival time to be an active antimalarial agent. The extract significantly prolonged the survival time of mice as compared to the control. However, unlike CQ, the mice were not cured. This might be due to the incomplete clearance of the parasite or short half-life profile of active constituents [16, 28, 29].

A candidate antimalarial agent should elicit parasite suppression of 30% or greater [39]. Hence, the extract at all doses tested was found to possess a promising chemosuppression effect and found to be active in counteracting the malaria parasite. Furthermore, if the antimalarial activity of a compound displayed a percent growth inhibition of ≥50% at a dose of 500-250, 250-100, and ≤100 mg/kg/day, the literature grades it as moderate, good, and very good, respectively [16, 39–41]. Therefore, the fruit of *C. frutescens* possesses a very good antimalarial activity.

It can be deduced from the acute toxicity test that the oral medial lethal dose (LD_50_) of the extract could be greater than 2000 mg/kg of the extract as per OECD guideline no. 425 [22]. Evidence from this data could serve as a justification for the safe folkloric use of the fruit of *C. frutescens* for the treatment of malaria by the local people in Ethiopia and Uganda [20, 22].

## 5. Limitations of the Study

Due to the lack of genetically modified immunocompromised mice, the study was not conducted on *P. falciparum* which is resistant to chloroquine. As *P. falciparum* cannot infect normal mice, it would be good if the extract is also tested against the actual resistant parasite. However, the study is ok as most malaria screening tests utilize *P. berghei* at the early stage of investigation.

## 6. Conclusion

The findings of the present study indicated that the fruits of *C. frutescens* have a promising *in vivo* antimalarial activity that can serve as a potential source to develop effective and safer antimalarial drugs. The highest chemosuppressive effect was exhibited by the upper dose of the tested extract indicating the presence of high concentration of bioactive principles in this dose. Moreover, the data would provide evidence to endorse the traditional use of the plant by the local communities for the treatment of malaria in Ethiopia and Uganda.

## Figures and Tables

**Table 1:** The 80% methanolic crude fruit extract of *Capsicum frutescens* consequences on parasite load and the length of survival of mice infected with *P*. *berghei*.

Group	% parasitemia	% suppression	Survival time (day)
CON	57.84 ± 1.48	0.00	6.25 ± 0.55
CFE100	15.82 ± 0.76	72.65^v^,x^,y^,z^^	17.66 ± 0.37^v^,y^,z^^
CFE200	7.01 ± 0.91	87.88^v^,w^y^,z^^	22.02 ± 0.51^v^,y^,z^^
CFE400	3.88 ± 0.85	93.29^v^,w^,x^,z^^	27.42 ± 0.74^v^,w^,x^,z^^
CQ10	0.00 ± 0.00	100.00^v^^	>30.00 ± 0.00^v^^

Values are described as mean ± SEM (*n* = 6). CON: negative control; CFE: crude fruit extract of 80% methanol; CQ: chloroquine base. ^v^Compared to CON; ^w^compared to 100 mg/kg; ^x^compared to 200 mg/kg; ^y^compared to 400 mg/kg; ^z^compared to CQ10; ^^^*p* < 0.05. In the first column, numbers next to letters (10, 100, 200, and 400) represent dose in mg/kg.

**Table 2:** Changes in weight and rectal temperature of mice infected with *P. berghei* and treated with the 80% methanolic crude fruits extract of *Capsicum frutescens*.

Group	Weight (g)			Temperature (°C)		
	D_0_	D_4_	Change	D_0_	D_4_	Change
CON	28.84 ± 0.65	25.24 ± 0.45	−3.60 ± 2.02	37.05 ± 0.19	35.01 ± 0.30	−2.04 ± 0.05
CFE100	29.12 ± 0.82	28.86 ± 0.42	−0.26 ± 0.14^v^x^y^z^^	37.02 ± 0.41	36.90 ± 0.37	−0.12 ± 0.01^v^x^y^z^^
CFE200	28.92 ± 0.71	28.82 ± 0.31	−0.10 ± 0.20^v^w^y^z^^	36.65 ± 0.37	36.62 ± 0.31	−0.03 ± 0.03^v^w^y^z^^
CFE400	29.21 ± 0.49	29.19 ± 0.23	−0.02 ± 0.15^v^w^x^z^^	37.13 ± 0.23	37.11 ± 0.25	−0.02 ± 0.02^v^w^x^z^^
CQ10	29.03 ± 0.80	29.33 ± 0.56	0.30 ± 0.05^v^^	36.91 ± 0.20	36.97 ± 0.18	0.06 ± 0.04^v^^

Values are described as mean ± SEM (*n* = 6). D_0_: pretreatment value on day 0; D_4_: posttreatment value on day four; CON: negative control; CFE: crude fruit extract of 80% methanol; CQ: chloroquine base. ^v^Compared to CON; ^w^compared to 100 mg/kg; ^x^compared to 200 mg/kg; ^y^compared to 400 mg/kg; ^z^compared to CQ10; ^^^*p* < 0.05. In the first column, numbers next to letters (10, 100, 200, and 400) represent dose in mg/kg.

**Table 3:** Preliminary qualitative phytochemical screening of the 80% methanolic fruit crude extract of *Capsicum frutescens*.

Phytochemicals	Test result
Alkaloids	∗
Tannins	∗
Saponins	∗
Flavonoids	∗
Terpenoids	∗
Steroids	x
Glycosides	∗

x: shows absence of phytoconstituents; ∗: shows presence of phytoconstituents.

## Data Availability

Vouchers and specimens of the investigated plant are deposited at the national herbarium of Addis Ababa University, College of Natural Sciences. The datasets used to support the finding of this study are available from the corresponding author upon request.
